# The SARS-CoV-2 pandemic: the race to trace: contact tracing scale-up in San Francisco—early lessons learned

**DOI:** 10.1057/s41271-021-00285-y

**Published:** 2021-06-04

**Authors:** Michael Reid, Wayne Enanoria, Juliet Stoltey, Susan Philip, Jonathan Fuchs, Amy Lockwood, Elizabeth Krueger, Karen White, Jessica Celentano, George Rutherford, Susan Scheer, Trang Nguyen, Darpun Sachdev

**Affiliations:** 1 University of California, San Francisco, San Francisco, USA; 2 Population Health Division, San Francisco Department of Public of Health, San Francisco, USA; 3 Division of Infectious Diseases, UC San Francisco, 513 Parnassus Avenue, S380, San Francisco, CA 94143 USA

**Keywords:** COVID-19, Contact tracing, Epidemiology, Containment

## Abstract

In order to effectively control spread of coronavirus 2019 (COVID-19), it is essential that jurisdictions have the capacity to rapidly trace close contacts of each and every case. Best practice guidance on how to implement such programs is urgently needed. We describe the early experience in the City and County of San Francisco (CCSF), where the City’s Department of Health expanded contact tracing capability in anticipation of changes in San Francisco’s ‘shelter in place’ order between April and June 2020. Important prerequisites to successful scale-up included a rapid expansion of the COVID-19 response workforce, expansion of testing capability, and other containment resources. San Francisco’s scale-up offers a model for how other jurisdictions can rapidly mobilize a workforce. We underscore the importance of an efficient digital case management system, effective training, and expansion of supportive service programs for those in quarantine or isolation, and metrics to ensure continuous performance improvement.

## Key messages

- San Francisco succeeded in implementing a language-concordant COVID-19 contact tracing program designed to treat all equitably through rapid workforce mobilization, effective training, and prompt introduction of a digital case management platform.
- Rapid expansion of contact tracing capability required parallel expansion of COVID-19 testing and containment programs to ensure adequate provision of services to those in isolation and quarantine.
- Evaluating the epidemiologic dividend of COVID-19 contact tracing efforts demands consensus on appropriate indicators for assessing both programmatic process and outcome.

## Introduction

Before lifting mitigation efforts, it is essential that jurisdictions have capacity to rapidly test all priority populations for COVID-19 containment efforts, identify and isolate positive cases, and conduct contact tracing for close contacts of each and every case. Careful implementation of these key elements will enable loosening of social distancing measures. However, to undertake the unprecedented contact tracing required [1] will require a substantial augmentation of local public health workforces in the United States (US). We describe the early experience in the City and County of San Francisco (CCSF), between April 13 and June 8, 2020, where the San Francisco Department of Public of Health (SFDPH) rapidly expanded its contact tracing capability in anticipation of changes in San Francisco’s ‘shelter in place’ order [2]. While the epidemiologic impact of this expansion has been described in a separate manuscript [3] here we highlight important prerequisites to successful scale-up, including the role of force multipliers such as technological tools, effective knowledge management capabilities, parallel expansion of effective containment programs, and metrics to ensure continuous performance improvement. We highlight the importance of a culturally sensitive response to ensure that contact tracing efforts are maximally effective in reaching those populations most impacted by COVID-19.

## Methods

### Establishing the foundation: enhanced case investigation, case finding, and containment efforts during shelter-in-place

On February 25, 2020, San Francisco’s Mayor, London Breed, was the first city mayor in the United States to declare a state of emergency to mobilize financial resources to focus on COVID-19 preparedness [2]. On March 17, 2020, Mayor Breed responded to recognition of widespread community transmission and increasing public awareness of the need to ‘flatten the curve.’ In coordination with five other Bay Area counties and the City of Berkeley, she ordered San Francisco residents to shelter in place. By then SFDPH had determined that 58 people had fallen ill with COVID-19 and one had died in a city of 900,000 inhabitants [4], and the San Francisco Public Health Laboratory was performing approximately 260 RT-PCR tests per day [5]. The SFDPH Disease Prevention and Control Branch had already mobilized to respond to the epidemic, performing 150-200 case investigations a week by the time of the health order. Nonetheless, SFDPH recognized that swift action was imperative to prevent overwhelming transmission that would render more enhanced case investigation and contact tracing unfeasible [6]. The SFDPH Department Operations Center (DOC) leadership identified the need to enhance capability to implement a robust and expansive contact tracing. Moreover, DOC leadership recognized that to have maximal impact, contact tracing expansion needed to happen in parallel with other critical elements, including (1) increased testing, particularly for populations at highest risk for COVID-19 morbidity and mortality, (2) mobilizing a workforce to expand capability and implement case investigation and contact tracing, and (3) ensure holistic, social services to enable provision of adequate isolation and quarantine spaces for all those in need, ensuring equitable distribution to all.

### Increasing access to testing

The DOC Testing Branch scaled resources to increase case finding for all contacts of COVID cases. It responded to the need for mass testing to identify outbreaks in congregate settings, with particular focus on long-term care facilities, homeless shelters, and single-room occupancy hotels (SRO) [7]. By the end of April 2020, increased diagnostic capacity, in tandem with increased capability to collect more samples at more testing venues, meant that the city was able to broaden its testing criteria to include all close contacts of confirmed COVID-19 cases, without regard to symptoms (or absence of them) among contacts. As well as overseeing implementation of these key elements to meet the needs of the city’s entire population, SFDPH instituted plans to implement an ambitious plan to mobilize a workforce of contact tracers sufficient to address the anticipated increase in cases. SFDPH was able to achieve this with assistance from the University of California, San Francisco (UCSF) which supported training, analytics, and informatics with expertise and project management capability. Only in this way would San Franciso’s health authority be able to recommend loosening of shelter-in-place requirements.

### Mobilizing an expanding workforce

Recognizing that there was a pressing need to rapidly expand the workforce dedicated to contact tracing, SFDPH looked to other countries and epidemiologic modeling to estimate the number of individuals who would be needed to undertake contact tracing. Wuhan China mobilized 9000 personnel for a population of 11 million [8]; Canada activated 2700 persons to support its contact tracing effort [9]. The SFDPH contact tracing planning team estimated a need for 100–150 contact tracers for a population of approximately 900,000. To validate this estimate, our Case Investigation and Contact Tracing (CICT) management team, composed of public health experts from SFDPH and UCSF, projected potential needs based on various scenarios for how the number of incident cases and their contacts might increase over time. We also approximated the time it might take to perform contact tracing, even using efficient technological tools. Later, the CICT management team revised these initial projections by assessing how many cases and contacts the team reached in a given shift.

Given that SFDPH had already activated several members of its staff to work on COVID-related activities, the CICT management team discussed where else to find personnel for meeting this pressing human resource need. In partnership with the University of California San Francisco, SFPDH proposed a plan to rapidly train a workforce of medical students and retired clinicians, city and county (CCSF) librarians, and other civil servants who were unable to fulfill their normal duties under the shelter-in-place order. Training specific to their new responsibilities included ‘motivational interviewing’ (a directive, client-centered counseling style for eliciting behavior change) [10] and the confidentiality and privacy requirements of the CICT program, as outlined in the United States Health Insurance Portability and Accountability Act (HIPAA). The CICT training team, led by staff and faculty from UCSF, conducted most of the training on Zoom^®^; topics included disease transmission (basic information), principals of case isolation and quarantine for contacts, ethics of public health data collection, importance of cultural sensitivity, specifics of local processes and data collection, and characteristics of the San Francisco healthcare system.^1^ The training required completion by all participants of sections on confidentiality, privacy, and HIPAA compliance.

### Ensuring comprehensive support

**Fig. 1:**
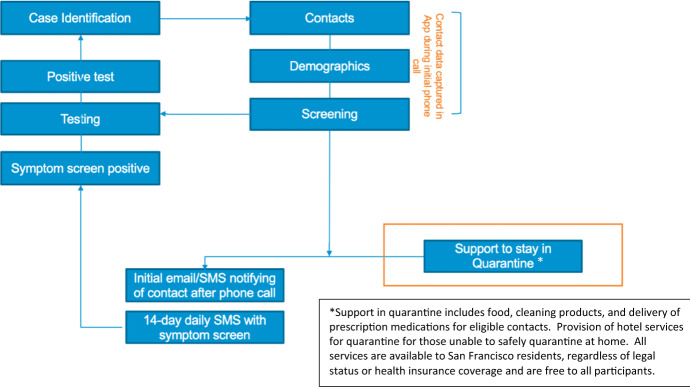
Current contact tracing workflow

The CICT management team repeatedly revised the contact tracing workflow to optimize client-centered care, team efficiencies, and data collection. (See Fig. 1.) SFDPH recognized a need for substantial support for many of those infected with COVID-19 (cases) and their contacts to safely isolate and quarantine. Thus, the DOC Containment Branch expanded capacity to isolate and quarantine all such people outside of their residences. The Containment Branch also made agreements with hotel owners and with a newly developed Internet-based bed management system (RTZ Systems, Lafayette California) in partnership with multiple CCSF departments, businesses, and community-based organizations. Through a coordinated program across several departments of city government, the DOC Containment Branch also arranged for food deliveries, financial resources, and cleaning supplies for all those eligible.

## Results

Between April 13 and 8 June 8, 2020, the 7-day moving average number of SARS-CoV-2 nucleic acid amplification tests performed in CCSF increased from 453 to 3295 [11]. During the same time interval, SFDPH identified 1633 laboratory-confirmed cases in non-outbreak settings. Of these, the SFDH case investigation team succeeded in reaching 1394 (85.4%). During the study period, the team traced 1214 close contacts, notified 1017 (83.8%), and tested 457 (37.6%).

To reach these contacts, the tracing workforce expanded from 19 personnel in the week of 13 April, to 118 personnel in the week of 8 June. Notably, the time from contact registration to first attempted contact dropped from approximately 5 days during the week of 13 April to less than 1 day by mid-May 2020.

## Discussion

Between April and June 2020, SFDPH was able to implement a robust contact tracing program to respond to COVID-19 pandemic. Effective contact tracing provided critical information to guide subsequent policy decisions about when it would be appropriate to move beyond requiring all residents to shelter in place.

We draw several key lessons from the experience.

First, to work effectively, the contact tracing workforce needed to be empathetic, culturally competent, focused on the needs of the city’s most underserved populations, and continually responsive to results. The program prioritized recruiting a workforce fluent in the languages of communities in San Francisco most impacted by COVID-19. Paramount was ensuring that language abilities of the workforce matched languages preferred by those affected, cases and contacts. The SFDPH contact tracing program sought partnerships with community-based organizations (CBOs) to facilitate ‘buy-in’ and participation from communities represented by the CBOs and to ensure the workforce included members of their communities.

Second, the CICT program quickly adopted a digital system to support ‘manual’ contact tracing. Because effective contact tracing requires identifying and listing contacts from each case and means to communicate with all contacts listed to inform each of potential exposure and link them to diagnostic resources and self-quarantine information and services, San Francisco needed a technological tool for customer relations management (CRM) and case management. After a rapid, but exhaustive review of digital options, SFDPH partnered with Dimagi (Boston, Massachusetts) to make use of their web-based COVID-19 tracking application, CommCare [12]. The CICT program chose this platform for the simplicity of its user-interface, its suitability for effective SMS monitoring of people on the contact list, and the potential for integrated visualization, and its analytic capabilities. This choice allowed SFDPH to maintain and manage the contact and case investigation databases, while adhering to CCSF’s strict confidentiality requirements mandated by US law (HIPAA). To enable communication between multidisciplinary teams for ensuring rapid notifications, optimizing testing referrals, and accommodating rapid linking of the disease outbreak teams and the contact tracing teams and workflows, we later added additional functions to the system. SFDPH subsequently transitioned to another digital platform, CalConnnect (Richmond, California). It aligned San Francisco with the majority of other jurisdictions in California and afforded the contact tracing team early and invaluable efficiencies. These include tracking of the average time from case interview to contact notification and week-by-week assessment of how quickly the program was reaching contacts. It also allowed us to stratify that process metric by language preference and race.

**Fig. 2:**
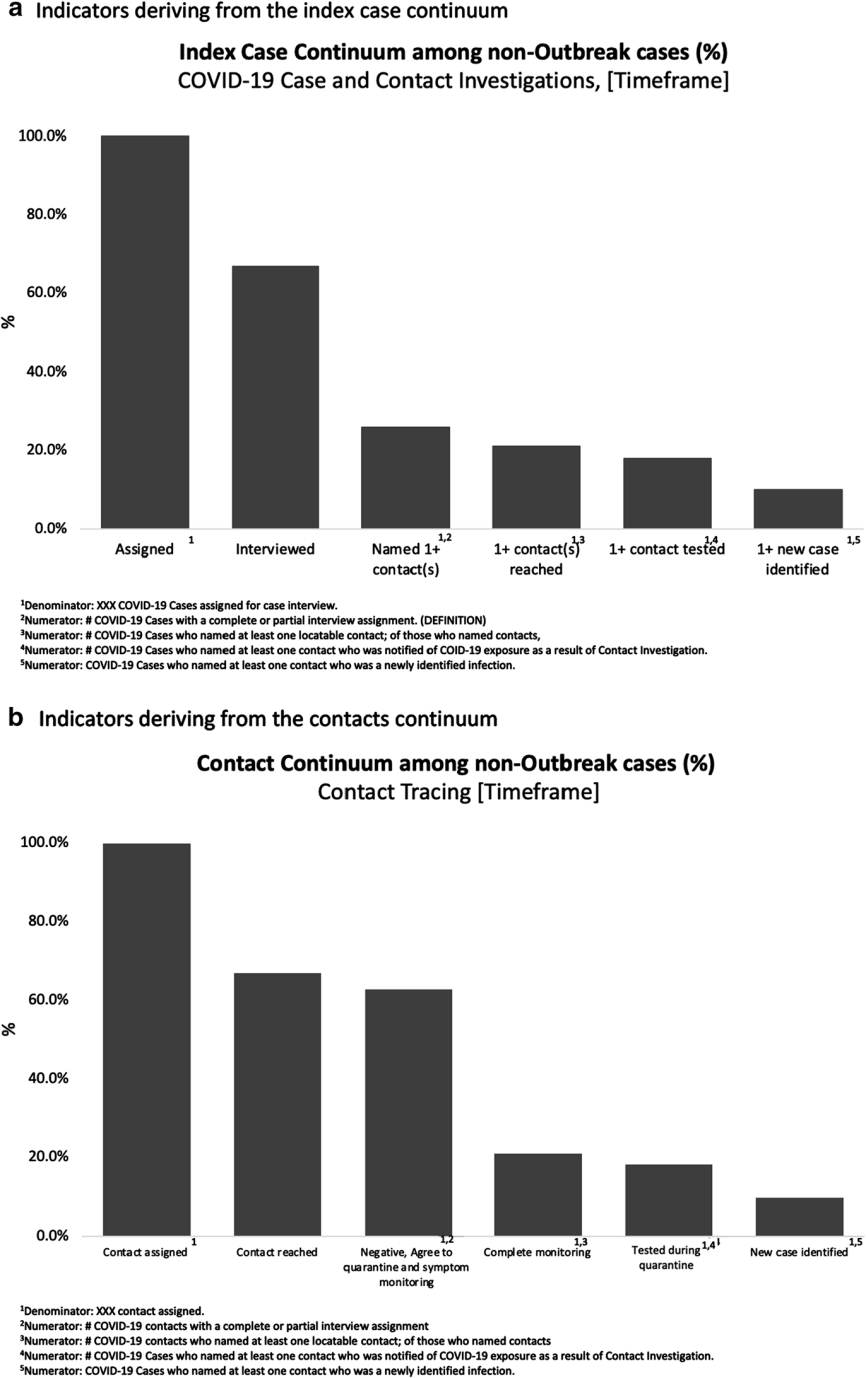
Illustrative COVID-19 contact tracing indicators. **a** Indicators deriving from the index case continuum. ^1^Denominator: XXX COVID-19 Cases assigned for case interview. ^2^Numerator: # COVID-19 Cases with a complete or partial interview assignment. (DEFINITION). ^3^Numerator: # COVID-19 Cases who named at least one locatable contact; of those who named contacts, ^4^Numerator: # COVID-19 Cases who named at least one contact who was notified of COID-19 exposure as a result of Contact Investigation. ^5^Numerator: COVID-19 Cases who named at least one contact who was a newly identified infection. **b** Indicators deriving from the contacts continuum. ^1^Denominator: XXX contact assigned. ^2^Numerator: # COVID-19 contacts with a complete or partial interview assignment. ^3^Numerator: # COVID-19 contacts who named at least one locatable contact; of those who named contacts. ^4^Numerator: # COVID-19 Cases who named at least one contact who was notified of COVID-19 exposure as a result of Contact Investigation. ^5^Numerator: COVID-19 Cases who named at least one contact who was a newly identified infection

Third, the program quickly adopted metrics for measuring impact. The CICT management team developed assessment tools similar to those used to measure the impact of syphilis and HIV contact tracing efforts. Primary measures of success included (1) number and percent of new cases interviewed, (2) number of new cases that identified at least one contact by name and provided contact’s phone number, (3) number and percent of cases with at least one contact reached, (4) number and percent of cases with at least one contact tested, and (5) number and percent of cases with at least one contact newly diagnosed with COVID-19, as illustrated in Fig. 2a. Related metrics allowed us to evaluate process outcomes of contact tracing efforts, including the proportion of all contacts interviewed, tested, and found to be positive (Fig. 2b). Fourth, the CICT management team sought to track and reduce time taken for and between each critical step in the process, time from: test performed to test result; test result to case interview; contacts elicited to initial interview of contact; and most critically, time until contacts’ started quarantine. The most critical time interval proved to be that from the index case test result to testing of the contact. Acting on this information allowed us to assure containment of the disease and close monitoring.

At the start of CCSF’s contact tracing program, it did not link to or incorporate any kind of a Blue-Tooth enabled smart phone exposure notification (EN) option. We had not considered using other data streams (such as credit card transaction data, mobile phone geospatial tracking information, or surveillance camera footage) to enhance precision of contact tracing activities applied in other settings [13–15]. While we recognize its potential role, no suitable EN technology had been validated to complement ‘manual contact tracing.’ Even though California Department of Public Health has subsequently developed plans to implement a phone-based EN application, CA Notify (California), success will require rigorous application of privacy protections and responsible management of data collected [16, 17]. Those designing the next generation of contact tracing efforts will need to assess to what extent EN tools enhance existing efforts.

## Limitations

A major limitation of this work relates to the challenges of assessing which interventions contributed most to COVID-19 containment. Many factors, including the expanding workforce and the ever-changing digital platform, contributed to the impact of the program; however, it was challenging to distinguish which of these factors was most impactful. Our analysis does not include any assessment of the epidemiologic impact of the program, nor of subjective experiences of those individuals reached by case investigation. Description of program implementation contains no assessment of CCSF’s programmatic response to COVID-19 surges that occurred after the initial scale-up. Nonetheless, the description does highlight programmatic priorities that remain consistent. These include (1) rapidly testing all contacts with minimum administrative constraints and without any financial barriers, (2) improving the efficiency of strategies to elicit the names of all close contacts from those diagnosed with COVID-19, and (3) enhancing the scope and effectiveness of a comprehensive set of services for those needing to isolate or quarantine to assure support for all contacts, especially those with the fewest resources, to remain in quarantine for as long as necessary.

## Conclusion

The San Francisco model for contact tracing—using a digital tool to mobilize and rapidly train a much expanded workforce—offers a model relevant to other health authorities, both in the United States and beyond. The approach allowed for effective contact tracing without undermining public concerns about non-consensual use of data. Our experience highlights the importance of strategic management, training and support for this workforce, and technology as a workforce multiplier. The San Francisco experience also teaches us that contact tracing is not a ‘silver bullet.’ Contacts cannot be expected to safely self-isolate or self-quarantine without a comprehensive set of supportive services. Contact tracing is a prerequisite of a robust COVID-19 containment response, but it will be futile unless implemented as part of an expansive public health vision incorporating all activities needed to control spread of COVID-19, especially targeting the vulnerable and underserved populations that have been most impacted by the pandemic.

## Footnotes

1 All of these training resources are available at https://trailhead.salesforce.com/en/content/learn/trails/get-started-as-a-contact-tracer.
